# Editorial: Genotype-Phenotype Correlation in Parkinsonian Conditions

**DOI:** 10.3389/fneur.2021.743953

**Published:** 2021-08-23

**Authors:** Luca Marsili, Ignacio F. Mata, Marcelo A. Kauffman

**Affiliations:** ^1^Department of Neurology, James J. and Joan A. Gardner Center for Parkinson's Disease and Movement Disorders, University of Cincinnati, Cincinnati, OH, United States; ^2^Genomic Medicine, Lerner Research Institute, Cleveland Clinic Foundation, Cleveland, OH, United States; ^3^Consultorio y Laboratorio de Neurogenética, Centro Universitario de Neurología José María Ramos Mejía, Buenos Aires, Argentina

**Keywords:** genetic parkinsonism, sporadic Parkinson's disease, LRRK2, GBA, genotype-phenotype correlation

With the diffusion of cost-effective genetic analyses, an increase in the spectrum of reported genetic variants associated with sporadic Parkinson's disease (sPD) (e.g., glucocerebrosidase—*GBA*) and monogenic parkinsonisms (dominant, recessive, and atypical forms) has been achieved. Each single variant may be associated to distinct prominent phenotypic characteristics helpful for diagnostic and prognostic purposes, thus ushering the era of precision medicine for movement disorders (1). This special issue was designed to explore the genotype-phenotype correlation of parkinsonian conditions extensively. Of the 18 papers initially submitted to the journal by international researchers, 14 were considered suitable for publication after a thorough peer-review process. These included three original research articles, five reviews, five brief research reports, and one mini-review. The following is a short summary of the main results of each of these manuscripts.

Guadagnolo et al., provide a detailed review of genotype-phenotype correlation in monogenic parkinsonisms. Their clinical presentations may range from cases indistinguishable from sPD to very early-onset (childhood) conditions with several associated neurological features. Despite the broad clinical spectrum and the multiple genes involved, the phenotype of these conditions is strictly related to the altered cell function and inheritance pattern. Genotype-phenotype studies in genetic parkinsonisms may help in the earlier identification and in the development of disease-modifying treatments based on precision medicine strategies. Menozzi and Schapira, in their mini-review, focus on the genotype-phenotype correlation of *GBA*, one of the most important known risk factors for sPD (2). *GBA*-PD patients show more severe motor and non-motor symptoms, rapid disease progression, and reduced survival than non-carriers. However, the impact of *GBA* variants on clinical phenotype may significantly vary. Homozygous, compound heterozygous, and the “complex” (recombinant) and “severe” heterozygous variants' carriers display aggressive phenotypes with faster disease progression. Differently, the so-called “mild” and “risk” (not causative) variants have slower and more benign disease courses. The stratification of *GBA* carriers in the prodromal and manifest phase of PD is fundamental for patients' counseling, prognosis, and a better understanding of the possible efficacy of advanced treatments.

The disease spectrum of genetic parkinsonian conditions is constantly expanding. Salles et al., reviewed the full spectrum of clinical manifestations of *ATP1A3* mutations. Pathogenic variants in this gene have been implicated in several phenotypes: (a) rapid-onset dystonia-parkinsonism; (b) alternating hemiplegia of childhood; and (c) cerebellar ataxia, *pes cavus*, optic atrophy, and sensorineural hearing loss (CAPOS syndrome). Since the original descriptions of these disease entities, a growing number of cases showing overlapping features have been reported. We can consider *ATP1A3*-related disorders as a clinical continuum rather than distinct entities, with clinical features ranging from early-life epilepsy (in the most severe extreme side of the spectrum) to rapid-onset dystonia-parkinsonism (in the milder side of the spectrum). Lesage et al., investigate the clinical variability of *SYNJ1*-associated early-onset parkinsonism. They describe two cases carrying previously unreported biallelic mutations of *SYNJ1* and two siblings with the recurrent homozygous p.R258Q mutation. The patients studied show different clinical symptoms ranging from epilepsy, intellectual disability, and progressive parkinsonism (biallelic mutations) to a parkinsonian syndrome with no atypical features and slow disease progression (p.Y832C mutation).

The most frequent forms of autosomal dominant monogenic parkinsonism are those related to mutations in the *LRRK2* gene (3). Vinagre-Aragón et al. in their review, explored the role of the *LRRK2*-R1441G variant in the Basque population. These cases appear to be associated with a homogeneous, less aggressive, “pure” motor phenotype, which may resemble sPD. Genetics constitute a relevant aspect, as it may significantly influence the phenotype, with differences according to the mutation within the same gene, not only in familial but also in sPD. Thus, expanding our understanding of genetic parkinsonisms implies an extension of knowledge regarding sPD, which may be relevant for future therapeutic implications of all parkinsonisms. Leija-Salazar et al., in their original research article investigate single nucleotide variants (SNVs) in brains from patients with sporadic synucleinopathies and a monozygotic twin carrying the *LRRK2*-G2019S variant. Somatic SNVs in coding regions of genes associated with synucleinopathies could contribute to these disorders, according to the number of affected cells and mechanisms of diffusion of the causal agent (4). The authors did not detect disease-relevant somatic SNVs, although their presence at the initial stages of neurodegeneration is postulated. These results suggest that, while coding somatic SNVs in neurodegeneration are rare, other somatic variants may have a pathogenic role in synucleinopathies.

Genome-wide association studies (GWAS) have suggested the possible role of several genetic variants as risk factors for the development of sPD (4–6). Szwedo et al., explore the impact of *SNCA* polymorphisms on a large longitudinal population-based cohort of sPD patients. Their results show that the variant rs356219 has a minor effect on modifying disease progression, whereas no differences were associated with other variants. These results imply that *SNCA* variants associated with sPD risk are not central factors responsible for the clinical heterogeneity of sPD. Tunold et al., describe a significant effect of *APOE* and *MAPT* genetic variants on dementia in pathologically confirmed sPD. These results support the critical role of *APOE*-ε4 and *MAPT*-H1 haplotypes in the etiology of sPD-related dementia, with potential relevance for patient selection for future clinical trials of disease-modifying therapies. Moran et al., explore the motor, cognitive, psychiatric, and olfactory functioning between carriers and non-carriers of *GBA* variants (both groups without PD). *GBA* mutation carriers show reduced performance on executive functioning, and carriers with hyposmia have lower cognition scores than those without hyposmia. Hence, pre-manifest features of PD may not be found in *GBA* mutation carriers; however, a difference in executive functioning among some non-manifesting *GBA* mutation carriers is present. These findings, combined with hyposmia, should be further investigated as possible biomarkers for pre-manifest and pre-clinical *GBA*-related sPD. Markopoulou et al., analyze single nucleotide polymorphisms (SNPs) identified in previous GWAS studies, together with low frequency and rare variants at parkinsonism-associated genes from the MDSgene database, in sPD patients. They suggest that genetic risk factors for sPD may variably affect the phenotypic presentation and that genes associated with sPD risk are also differentially associated with individual phenotype at baseline.

Torrealba Acosta et al., and Milanowski et al., investigated monogenic parkinsonism-associated variants in some underrepresented populations. Torrealba Acosta et al., studied the genetic and clinical underpinnings of PD patients of Costa Rican origin. Although they do not identify a direct relationship between the genes tested and PD, they find six rare *LRRK2* variants located in the C-terminal-of-ROC (COR) domain, non-synonymous *GBA* variants (p.T369M, p.N370S, p.L444P) in three healthy individuals and one PD patient carrying a pathogenic *GCH1* variant (p.K224R). They also show that physical activity and education are correlated with better motor and cognitive outcomes, respectively, while hallucinations, falls, mood disorders, and coffee consumption correlate with worse cognitive performance. This is the first study clinically and genetically characterizing a cohort of Costa Rican PD patients, thus expanding the genomic research in the Latino population. Milanowski et al., screen 109 patients with PD from Nigeria finding 22 variants [*PRKN*, 9 (40.9%); *PINK1*, 10 (45.5%); and *DJ-1*, 3 (13.6%)]. They identify three (13.6%) rare, non-synonymous variants, without finding any homozygous/compound heterozygous carriers. This study underlines how, although more studies are needed in sub-Saharan African countries, population-specific variation may contribute to a better knowledge of the genes involved in PD in the local population and also to the interpretation of results from larger studies performed in European or Asian populations.

Finally, several lines of evidence have recently suggested a possible role of inflammation and lysosomal activity on the pathogenesis of parkinsonian conditions (7–9). Magnusen et al., review data from genetics, immunology, and *in vivo* and *ex vivo* functional studies showing that genetic defects associated with monogenic parkinsonisms might contribute to microglial cell activation and generation of pro-inflammatory cytokines and chemokines, responsible for neurodegeneration. Understanding the involvement of various immune mediators, their source, and the target could provide a better knowledge of PD pathogenesis for diagnostic and prognostic purposes. Abe and Kuwahara, review the roles of parkinsonian gene products implicated in the lysosomal pathway, namely *LRRK2, VPS35, ATP13A2*, and *GBA*, providing an overview of their disease-associated functions and their cooperative actions in the pathogenesis of sPD, based on the evidence from cellular and animal models. This study suggests that targeting lysosomal activation could represent a possible strategy to treat neurodegeneration.

In conclusion, the editors wish to thank all the authors, the reviewers, and the editorial board members for contributing to this special issue. We hope that this special issue might inspire future and novel research approaches in the genetics of parkinsonian conditions.

## Author Contributions

All authors listed have made a substantial, direct and intellectual contribution to the work, and approved it for publication.

## Conflict of Interest

The authors declare that the research was conducted in the absence of any commercial or financial relationships that could be construed as a potential conflict of interest.

## Publisher's Note

All claims expressed in this article are solely those of the authors and do not necessarily represent those of their affiliated organizations, or those of the publisher, the editors and the reviewers. Any product that may be evaluated in this article, or claim that may be made by its manufacturer, is not guaranteed or endorsed by the publisher.
